# Universal Capacitance Model for Real-Time Biomass in Cell Culture

**DOI:** 10.3390/s150922128

**Published:** 2015-09-02

**Authors:** Viktor Konakovsky, Ali Civan Yagtu, Christoph Clemens, Markus Michael Müller, Martina Berger, Stefan Schlatter, Christoph Herwig

**Affiliations:** 1 Institute of Chemical Engineering, Division of Biochemical Engineering, Vienna University of Technology, Gumpendorfer Strasse 1A 166-4, 1060 Vienna, Austria; E-Mails: vkonakovtuwien@gmail.com (V.K.); civan.yagtu.tuwien@gmail.com (A.C.Y.); 2 Boehringer Ingelheim Pharma GmbH & Co. KG Department Bioprocess Development, 88400 Biberach, Germany; E-Mails: christoph.clemens@boehringer-ingelheim.com (C.C.); markus_michael.mueller@boehringer-ingelheim.com (M.M.M.); martina.berger@boehringer-ingelheim.com (M.B.); stefan.schlatter@boehringer-ingelheim.com (S.S.)

**Keywords:** CHO cell culture, capacitance, fed batch, PLS, statistical model

## Abstract

Capacitance probes have the potential to revolutionize bioprocess control due to their safe and robust use and ability to detect even the smallest capacitors in the form of biological cells. Several techniques have evolved to model biomass statistically, however, there are problems with model transfer between cell lines and process conditions. Errors of transferred models in the declining phase of the culture range for linear models around +100% or worse, causing unnecessary delays with test runs during bioprocess development. The goal of this work was to develop one single universal model which can be adapted by considering a potentially mechanistic factor to estimate biomass in yet untested clones and scales. The novelty of this work is a methodology to select sensitive frequencies to build a statistical model which can be shared among fermentations with an error between 9% and 38% (mean error around 20%) for the whole process, including the declining phase. A simple linear factor was found to be responsible for the transferability of biomass models between cell lines, indicating a link to their phenotype or physiology.

## 1. Introduction

### 1.1. Problem Statement

One of the most important parameters in microbial or mammalian cell culture is the viable cell concentration (VCC). It is permanently subject to change in a typical batch or fed-batch process. VCC is usually measured offline by a cell counting device, but optimally, VCC could be also modeled without taking sample, *i.e.*, by an inline capacitance probe. The measurement principle of a capacitance probe is frequency-dependent polarization of dielectric material which enables the detection of the living spherical cells in form of capacitance [1,2,3,4]. This task is made more difficult when particles of varying shape and size are co-measured, which is the case when cells die and fragment. However, multivariate approaches are useful to filter out the noise [5,6] and can be used to model VCC during the whole process time instead of stopping when viability drops.

### 1.2. State of the Art

The models which describe the relationship between capacitance and VCC, are often based on linear regression, multiple linear regression, Cole-Cole and PLS. Applications of permittivity measurements with single and multi-frequency measurements were well summarized by Yardley *et al.* [7]. Noll and Biselli used linear regression to set a particular feed rate based on a constant glutamine consumption in continuous cell culture [8]. Zeiser [9] and Ansorge *et al.* [10] used multi-frequency permittivity measurements to monitor process events such as timepoint of infection and virus release in Sf-9 cells (with baculovirus), and later also in HEK cells (with lentivirus) [11]. While multivariate frequency measurements are more informative than classical single or dual frequency measurements, they are also much harder to interpret [1]. Opel *et al.* [5] described various methods to correlate VCC to capacitance in batch and fed-batch in great detail; in brief, linear models required frequent recalibration, Cole-Cole models were reliable during the cell growth phase, but not in other phases, and PLS models were accurate during the whole process phase, but required robust calibration datasets and complex analysis. There seems to be a general lack of motivation to publish data of the predictive accuracy of any capacitance model for a long-term mammalian cell cultivation. We hypothesize that one of the reasons for this may lie in the difficulty of standard models to describe the declining phase of a culture sufficiently well, as already mentioned by Cannizzaro [12]. Once the monitoring of cell concentration is accurate enough, control, in the form of closed-loop feeding strategies, might be employed to push cell count and titers in the process [13,14,15,16,17].

Conclusively, multivariate frequency models which were built with PLS regression from capacitance measurements had one particular benefit over all other models: They allowed the modeling of VCC also in the declining phase, implying that recalibrations may not be required. Instead, the transformed signal can be used for the whole process phase of a dynamic fed-batch.

### 1.3. Novelty of This Approach

PLS models of multivariate signals usually result in very low errors when created offline, *after* the process is over. These low estimation errors can become unacceptably high if the same model is applied as it is for the next process. The knowledge of what to include into the model before its construction is very important, as it will determine both reusability, transferability and with it the overall benefit of the signal.

Capacitances measured at high frequencies correlate with particles which are smaller than the viable cells [12,18]. In a statistical model, such as PLS, every single frequency is mean centered or standardized before calculating the coefficients and therefore has a certain weight in the analysis [19,20]. Particles (cell debris) may be detected and interpreted as biomass if those high frequencies are included. Capacitance maps of a multi-frequency scan can help to identify these variables, which must be excluded in order to generate a robust model. Afterwards, the same PLS model can be re-used for estimating VCC of a new clone by just adapting the model’s slope. This made the constructed PLS models so remarkably universal that they could be successfully applied from one lab in Germany to another one in Austria.

Conclusively, we found the combination of two points to be decisive for a transferable model based on capacitance: (i) variable selection *before* and (ii) slope adaptation *after* model construction. The knowledge of VCC in the system without the need of sampling unlocks radical new control strategies and concepts for improving cell culture bioprocesses in both process development and manufacturing.

### 1.4. Goal

The goal of this work is to establish a *transferable* VCC estimation model in CHO cell lines regardless of clone, setup or scale.

### 1.5. Roadmap and Workflow

**Figure 1:**
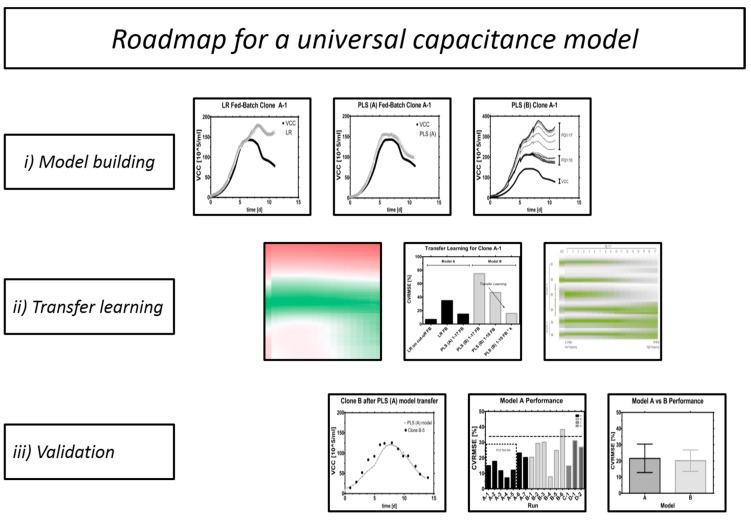
Roadmap.

We propose a novel method to use capacitance spectra for estimating biomass without further operator intervention in mammalian fed-batches. Typically, linear regression methods with just one frequency subtracted with the highest frequency (so-called “dual mode” to remove the influence of medium, *etc.* on measurement) are often enough for many short-term cultivation formats such as batches or short fed-batches while viability is still very high. However, continuous cultures and perfusion processes, where interfering cell debris is flushed out and cells are kept in a highly viable state for an extended period can also be described well with linear models [12,21]. In this work, we have found that PLS models are the ones which fit best to fed-batch formats, where viable cells undergo a dynamic growth and decline phase. Usually, useful statistical models depend strongly on high-quality historical datasets. Our approach was the transfer of *already existing* models instead of constructing new ones. Finally, we demonstrate that a transfer of the same model is not only possible between different clones but also between different scales. For this reason a suitable acceptance criterion was tested. Hence, this is a universal model for biomass estimation relying solely on real time information (Figure 1).

## 2. Experimental Section

### 2.1. Process Setup

Different CHO cell lines (A, B, C, D) producing different mAbs were cultivated in shake-flasks (Corning Incorporated, Corning, AR, USA) in incubators (Minitron, Infors, Bottmingen, Switzerland) with 5% partial CO_2_ pressure at physiological temperature (35–37 °C) on orbital shakers at 120 rpm (orbit 50 mm). Passaging was performed every third day in proprietary serum-free media and the bioreactors were inoculated at an initial seed density of (3–10) × 10^5^ cells/mL. At Vienna Technical University, different bioreactors (2.0 L operating volume, Infors HT, Bottmingen, Switzerland) were used than at Boehringer Ingelheim (Ingelheim, Germany) (2.0–80 L operating volume, no information on manufacturer).

#### 2.1.1. Data Source

Mammalian fed-batch fermentations using a capacitance probe (Biomass Sensor, Hamilton Bonaduz AG, Bonaduz, GR, Switzerland) in scanning mode were used to construct various linear and multivariate models. The bioprocesses were subject to variations in scale (2–80 L), internal bioreactor architecture (including different impeller types, H/D ratios and glass or stainless steel shell), setpoints of pH and pO_2_ (7.0 ± 0.2 resp. 5%–60%) (Hamilton Bonaduz AG, Bonaduz, GR, Switzerland), stirrer cascades, bolus additions of concentrated feed solutions, media and feed composition (both chemically defined and serum-free, proprietary formulations) and feed strategy. Temperature was held constant in all processes at 37 °C.

Capacitance measurements were recorded in mammalian bioreactor runs varying in scale, cell line and bioreactor geometry between sites. A summary of different setups can be seen in Table 1. On the top row, the individual experiments are discriminated against scale in columns, the second hierarchy is the cell clone in columns. The experiments are listed in rows. The first model (PLS-A) was created from the largest dataset using run A1–A5 and validated on runs A6–A7. The second model (PLS‑B) was created from B1–B3 and validated on B4–B6. After model transfer, PLS-A could be used for Clone B, C and D and Clone B could be used for Clone A, C and D, regardless of scale (see Results and Discussion section as well as supplement).

**Table 1:** Crosstable fermentation conditions.

Run	Scale 1 (80 L)			Scale 2 (2 L)		Chapter
	*Clone A*	*Clone B*	*Clone C*	*Clone B*	*Clone D*	**Finding the best model**
A1	**x**					
A2	**x**					
A3	**x**					
A4	**x**					
A5	**x**					
A6	**x**					
A7	**x**					
B1		**x**				**Variable selection**
B2		**x**				
B3				**x**		
B4	****			**x**		
B5				**x**		**Transfer learning**
B6		****		**x**		
C1			**x**			**Validation**
D1					**x**	
D2					**x**	

#### 2.1.2. Media

Media for the fed-batches are proprietary in composition and subject to variations in starting levels of metabolites, growth factors, *etc.* All components were serum-free and chemically defined.

#### 2.1.3. Cell Lines

All cultures were engineered CHO cells. Suspension of Clone B cells for experiments at the VUT were kindly provided by Boehringer Ingelheim (Ingelheim, Germany), while Clones A, C and D were used for model building and validation.

#### 2.1.4. Analytics

Process information was logged using the process management system Lucullus (PIMS, Lucullus, Biospectra, Schlieren, Switzerland). Capacitance spectra were recorded by exposing the medium broth via inline probe to an excitation frequency ranging from 0.3 MHz to 10 MHz every minute. The full capacitance spectrum (all 17 frequencies) was recorded but not all capacitance values were used to construct linear and multivariate models. Multivariate modeling was performed using Datalab software [21] (kindly provided by Prof. Lohninger, Vienna University of Technology, Vienna, Austria). The concentration of total cells, viable cells and viability was measured using an image-based white/dark classification algorithm after automatic trypan blue staining integrated in the cell counter (Cedex HiRes, Roche, Basel, Switzerland). Main metabolite concentrations (Glucose, Glutamine, Lactic acid, Glutamic acid, NH_4_, IgG) were measured on-line and off-line using a photometric robot (CubianXC, Optocell, Bielefeld, Germany). Amino acid concentrations were measured off-line by HPLC using pre-column OPA-derivatization (Agilent, 1100 HPLC, Santa Clara, CA, USA).

#### 2.1.5. Multivariate Data Analysis

The SIMPLS algorithm integrated in Datalab software was used to calculate all regression coefficients for any given model. These coefficients are finally multiplied with all or only a selection of available independent variables (capacitance measured at certain frequencies) to estimate the dependent variable (VCC). VCC (~20–80 data points) was interpolated offline but also in real-time when required, to match the very frequently measured variable capacitance (~500 data points) using the *spline* function (MATLAB) which preserves the curvy character of VCC in-between offline measurements. In this contribution, three Principal Components (PC) held over 99% of the whole variance while the remaining one percent held mostly noise and could be omitted from the analysis. All PLS models with more than three predictor variables (frequencies) were built with three PCs to predict VCC to make the models comparable. For the special cases that three PCs could not be used because fewer signals were used as input, the number of PCs had to be reduced to two resp. one (this was only the case where one or two frequencies were used for model construction). A more thorough explanation of constructing such a multivariate model [22,23,24,25] and its interpretation [19,26,27] is given elsewhere.

In total, 34 PLS models were constructed from Clone A data using standardization and mean centering, and 34 PLS models from Clone B data. From these 68 PLS models, 64 could be constructed with three PCs which made the models comparable. All models are also provided in the supplement and we want to invite the reader to test them with their own data.

(1) $$\hat{y}=a_{1}\cdot c_{1}+a_{2}\cdot c_{2}+a_{3}\cdot c_{3}\ldots +a_{17}\cdot c_{17}+d$$

In Equation (1), $\hat{y}$ represents the estimated VCC in (10^5^/mL) while $a_{1\ldots 17}$ are the individual capacitance values in (pF/cm) at a certain frequency (*i.e.*, 100 (pF/cm) at frequency 0.3 MHz) and $c_{1\ldots 17}$ are the regression coefficients for this frequency in (*i.e.*, −1.5 (cm × 10^5^)/(pF∙mL)), calculated by PLS-Regression. The intercept d of the model is given in the same units as VCC (10^5^/mL).

### 2.2. Acceptance Criteria and Control Specifications

The model’s root mean square error (RMSE) and its coefficient of variation (CVRMSE) were calculated for both the exponential phase and the whole process phase.

(2) $$RMSE=\sqrt{\frac{\sum \left(y-\hat{y}\right)}{n-1}}$$

(3) $$CVRMSE=\frac{RMSE}{\bar{y}}$$

In Equations (2) and (3), y represents the measured VCC in (10^5^/mL), $\hat{y}$ the estimated VCC in (10^5^/mL), and n is the number of observations and $\bar{y}$ represents the mean of the estimated VCC in (10^5^/mL) multiplied with 100 to obtain the CVRMSE in (%). All calculations are also available with examples in the supplement.

A robust model acceptance criterion that is still practical for purposes beyond process monitoring is simply not yet defined. Therefore, the following criterion is suggested to be acceptable from a process engineer’s point of view but will have to prove itself in practice.

#### CVRMSE

The CVRMSE does optimally not exceed 25% for the exponential growth phase and 33% for the whole process phase. An absolute upper limit to reject the model is defined at 50%. Model estimations which deviate between 33% and 50% CVRMSE are not rejected but it should be questioned if such deviations work for the given problem statement. Often, gross errors which include handling, liquid level, extreme process events, contamination, massive changes in large or small particle load in the broth and changes associated with cell morphology or membrane potential as well as flawed reference measurements (*i.e.*, after dilution or wrong classification between dead and viable cells) are the prime reasons for offsets [3,5,10].

The herewith suggested model acceptance may seem rather broad. However, the goal of this contribution is not running behind theoretical estimations which sound great on the paper but are absolutely unrealistic if any seemingly insignificant parameter during the process is changed. This contribution compares fermentations from process development where different media and feed formulations, reactor geometry in the same scale and between different scales and clones, process set-points and fermentation conditions with and without sudden feed additions and various process events interfere with regular fermentation profiles according to the book. Capacitance measurements are furthermore also sensitive towards other capacitors and signals affecting the measurement such as metallic probes, stirrers or different grounding in various scales and set-ups. The required offline analysis was performed by various operators working at different sites and although the protocols were the same, the same analysis can yield slightly different results when the devices or time between sampling and measurement are subject to variation.

It is absolutely necessary that this novel method holds its promise to perform well under authentic process conditions with often orthogonal process conditions as well as in standard processes, which are easier to maintain and evaluate. Other research groups used the capacitance signal with linear regression for control purposes [8], and modeled VCC with PLS using multiple frequencies *after* the process was completed, but a process where PLS with multiple frequencies was used to control a process has not been yet reported. We want to stress that using linear models requires offline calibration while PLS models could be calibrated once and used for the whole process time, if they are robust enough for the specified control purpose. Most contributions analyzed VCC with PLS models in hindsight and found low errors for this method, which might not tell the whole story, as the analysis method and selection of validation has a significant impact on the final error. What is often missing and therefore misleading is a realistic description of the performance of both linear and PLS models.

Our data suggests that the expected CVRMSE from LR models can be twice as high as CVRMSE in PLS models. The average CVRMSE from PLS models in this contribution was 21% ± 9% CVRMSE. Therefore, we are confident that the suggested criterion of maximum 33% CVRMSE is not only realistic, but also very suitable for this model and will find many applications in instances where VCC may be required as an input.

## 3. Results and Discussion

### 3.1. From Signal to Model

**Figure 2:**
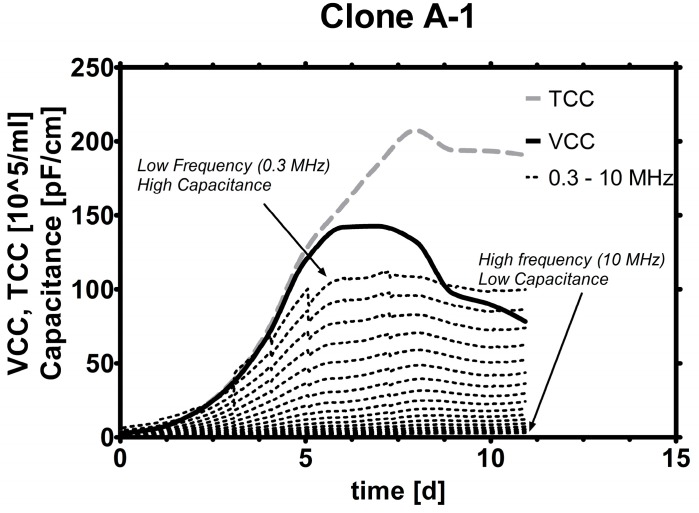
Signals over time: In this plot, VCC (solid line) and TCC (dashed line) are plotted together with capacitance values induced by varying frequencies (dotted lines). The lowest frequency (top dotted line) induces the numerically highest capacitance and reaches maxima around the time when VCC and TCC reach their maxima.

A typical capacitance spectrum is shown in Figure 2. A trend can be seen when capacitance measured at frequencies between 0.3 and 10 MHz is plotted together with viable cell count (VCC) and total cell count (TCC) over cultivation time. However, the trend of total cell count does not match as well as the one from VCC after cells start dying, which can be explained by the measurement principle of this probe [28]. Viable cells are able to sustain an intact cell membrane and build up capacitance when charged by a specific frequency, while dead cells, which have lost the ability to keep charged ions in their cytoplasm, do not contribute to capacitance in the same frequency range. This means that in theory, the probe is suited to detect viable cells very selectively.

### 3.2. Finding the Best Model

**Figure 3:**
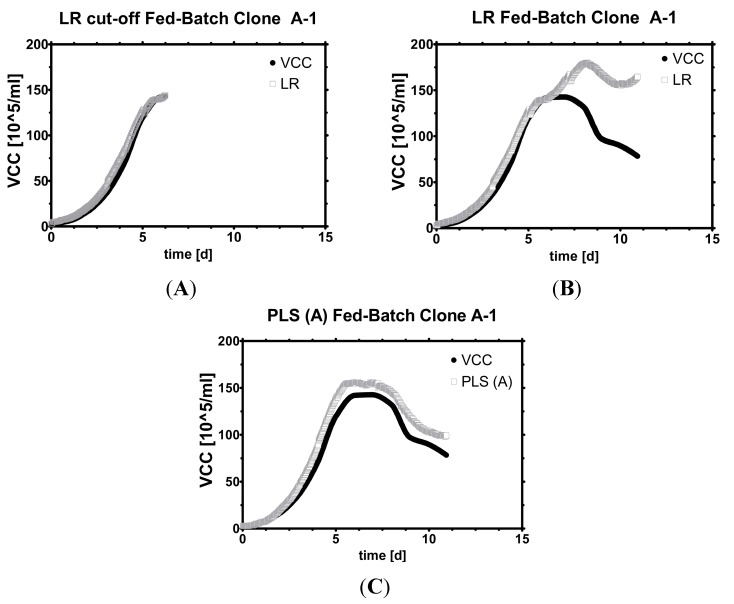
Model development. Fit *versus* measurement: (**A**) (cut-off) Fed-Batch while viability was high with a univariate model; (**B**) (full) Fed-batch with a univariate model; (**C**) (full) Fed-batch with a multivariate model.

As the first step, linear models were constructed for one Clone (A), scale (1) and fed-batch (A-1). Various phases were compared (highly viable fed-batch, full fed-batch in Figure 3) and discrepancies in performance found (see supplement for more data). As univariate models were not able to describe the declining viable cell concentration sufficiently, multivariate models were employed to find yet overseen correlations by weighting the coefficients of capacitance signals differently. As seen below, the multivariate model was best suited to predict VCC in a typical fed-batch cultivation.

#### 3.2.1. Linear Model

Linear models can be employed in all batch-type and even short fed-batch processes, *i.e.*, in virus production to seed trains for inoculation of production scale bioreactors [29,30]. Which frequency works best in describing VCC is somewhat disputable. Yardley [7] has summarized frequently used frequencies for mammalian cultivations which range from 0.5 to 0.8 MHz and a potential extension of up to 3 MHz. Our own observation is that in standard short-term fermentations, even the highest frequencies (~10 MHz) correlate excellently with VCC (R^2^ > 0.99, see supplement). Therefore, the standard frequency provided by the manufacturer was chosen (around 1 MHz) for LR (Figure 3A).

The trouble with linearly estimated VCC begins when cells leave the exponential growth phase (Figure 3B) in fed-batch cultivations. To be more precise, the calibrated probe mostly overestimates viable cell count, sometimes by a constant offset, other times accompanied with one or more spikes (Figure 3C). Thus, a linear calibration model can always be used before VCC peaks and only with great care or recalibrations later on. Whether VCC rises or falls is exactly the information one seeks in a fed-batch, and is very hard to answer in real time with only one single frequency, no matter which one was chosen.

Some authors therefore proposed to make use of an adapting frequency which is dependent on several cell-specific factors and can change during the course of a fermentation. In brief, this so-called critical frequency *fc* shall explain the deviations in a mechanistic way by pricing-in influences ranging from cell size change, conductivity and others [7,31,32], thus extending the regular linear model by several terms and parameters for which a mathematical fit in a new model (Cole-Cole) is required. Although the concept is without a doubt plausible, all fitted coefficients require calculation, pre-smoothing steps of the data and might differ under different process conditions, reactor geometries, physico-chemical conditions and so on. The factors might therefore require readjustments after every change of the bioprocess which might be more problematic in bioprocess development runs than in established process formats. This dataset was recorded in development format and therefore subject to severe disturbances where the spectra were very difficult to interpret. Because of transferability concerns, the development of mechanistic models is acknowledged [10,33,34,35] but not pursued in more detail.

#### 3.2.2. Multivariate Model

By using all frequencies instead of just one, these challenges are accepted by employing statistical multivariate models such as Multiple Linear Regression (MLR) or Partial Least Squares Regression (PLS or PLSR) to predict the target variable VCC more accurately. Other possible methods would encompass Principal Component Regression (PCR) [25], or Artificial Neural Networks (ANN) [36]. MLR (and also PCR) models tend to overfit any given dataset if enough data are available [20], while constructing an ANN was out of scope of this contribution, which should demonstrate the transferability of one designated model. Therefore, a PLS model was constructed with five runs of cell line Clone A and fit to the remaining two runs. The fit had an R^2^ > 0.95 and was useful for estimating VCC under the same conditions (Figure 3A,B). Similar results were found for Clone B constructed in two different scales as seen later on in this contribution.

### 3.3. Multivariate Variable Selection

#### 3.3.1. Scaling

Two methods were used to process capacitance data: Standardization (SD) and Mean Centering (MC). The difference is explained well in literature [37,38]. In general, MC seems to be used more frequently to construct PLS models.

The interpretation of coefficients derived from a statistical model could lead to an over-interpretation of the results. All capacitance signals alone correlate naturally positively with VCC, but due to the model fit some variables are finally attributed to a negative VCC trend which seems to not comply with the measurement principle. This may also be the reason why PLS models are readily created, but in contrast to linear regression the coefficients are actually never explicitly shown [5,12,39].

The final weighting procedure in PLS represents and considers also other effects in the course of the fermentations which are not immediately recognized. For instance, in our experiments, almost all multivariate models of this contribution correlate capacitance at a high frequency with a decline in VCC. When the highest frequency is removed from the model, the next-highest frequency takes the former’s role until in a linear model only one frequency is left (as included in the supplements).

It is hypothesized that this is probably caused by late-stage discrimination between VCC and charged, small particles, namely cell fragments and partially organelles, which store electric charge in form of capacitance and perhaps the main reason why this kind of model performs so well. Cannizzaro used the non-viable part to build a PLS calibration model from only dead cells [12]. The quantification of particle count proved to be so extremely difficult (CVRMSE between 33% and 141% in the calibration set and 77%–192% in the validation) that the information is of rather qualitative than quantitative nature. The main question, whether the model coefficients for only viable cell count are so robust that they are also valid for other systems becomes apparent when a transfer between clones and scales is demonstrated.

#### 3.3.2. Capacitance Maps

Ideally, there are no other dielectric materials except the viable cells, but in practice, a fermentation broth contains also dead, apoptotic, enlarged or fragmented cells and particles [8]. A heat map is constructed, where the individual capacitance values are represented as colors. These herewith called “capacitance maps” can give the operator a visual information of the distribution of dielectric material before a model is constructed.

**Figure 4:**
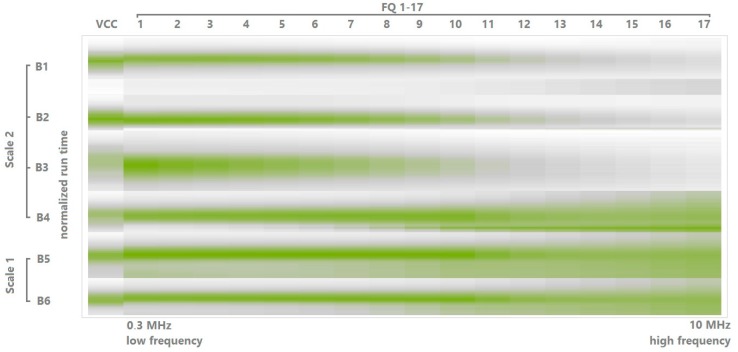
Normalized capacitance map: Normalized VCC are located on the very left, normalized capacitances in all remaining columns. High (left) and low (right) capacitance at different frequencies (FQ 1–17, from 0.3 to 10 MHz). The colors are relative to all runs, which means the maxima of all runs are green and the minima are white. Different scales are indicated. Frequencies which would later negatively influence the estimated VCC in the model can be thus identified visually. The region of interesting frequencies for model transfer encompasses frequencies 1:10.

Clone-specific capacitance maps were used to elucidate which frequencies are suitable for the model. The capacitance map displays VCC on the very left column, the time course of the fermentation follows from top to bottom in every column. The numerically largest signals can be extracted from low frequencies (on the left, starting with 0.3 MHz), while very high frequencies (on the right, 10 MHz) were numerically small, carrying almost no information except mostly noise. However, if the values are normalized, an interesting picture is obtained; different reactor geometries and cultivation conditions were found to have a significant impact on the capacitance fingerprint profile as can be seen in Figure 4. More data are available in the supplement. The new, potentially transferrable PLS models were constructed using only frequencies between 0.3 and 2.16 MHz. These new specifications were further tested in different clones, setups and scales.

### 3.4. Transfer Learning

A change leading to new measurement conditions, be it through a slight modification in substrate levels [1,4] or other modifications of the environment [34,40,41,42], leads to a common problem in chemometrics: The direct application of the model is no longer possible [43]. Finding a way to model the target variable under the new measurement conditions using either new calibration data or a new calibration model by transfer of knowledge that has already been learned is called transfer learning [44]. In this contribution, the concept of transfer learning was used by applying previous knowledge from simple linear regression to a more complex PLS model. To our knowledge, no multivariate biomass model was transformed successfully in such a way before. It is hypothesized that the new, multivariate signal behaves similarly to the univariate capacitance signal, but without the noise in the data. These multivariate models are potentially more useful than univariate models and in addition universally shareable.

#### 3.4.1. Direct Model Transfer

**Figure 5:**
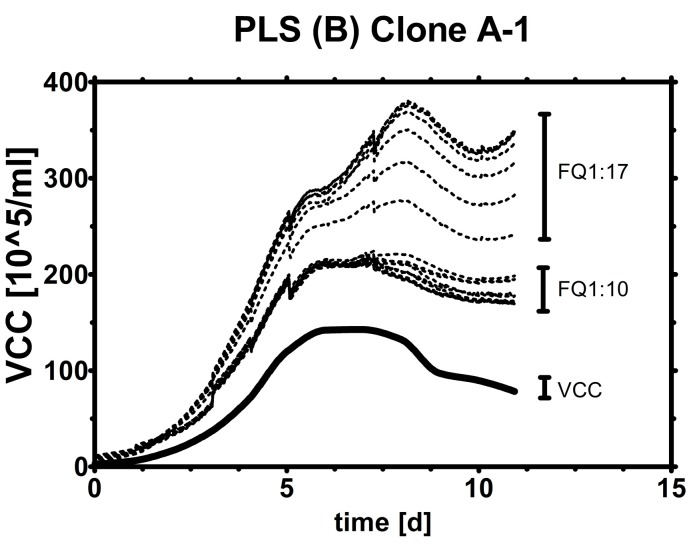
Testing frequency range effect on transferability. Model B is directly used on Clone A data with decreasing frequency input before PLS (B) model construction (1:17 all 17 frequencies, 1:10 frequency 1–10, *etc.*).

A cross-validation of PLS models was not performed because it is clear that any coefficients eventually will be found which fit to the given data. The effect of overfitting coefficients can be seen in Figure 5. Such coefficients might show their best performance for very similar runs under the same conditions (same clone or scale) but will not be able to estimate VCC under different conditions (*i.e.*, for a different clone in a different scale). This leads to the lengthy development of a plethora of small, local models. The extent of variation is visualized by using several PLS (B) models for one run (A-1). All Clone B models overestimate Clone A’s VCC but the fit gets better as certain high frequencies are excluded (Figure 5). After the partial success with the first direct transfer of Clone A to B and B to A models, various PLS models (A) and (B) as well as processing techniques were tested on run A-1 (see supplement).

#### 3.4.2. Attenuation Factor κ

After considering only selected frequencies, the fit suffers from less noise. Linear regression analysis of PLS estimation *vs*. measured VCC reveals that, *i.e.*, 1 unit estimated VCC corresponds to 0.69 units of real VCC with a very high R^2^ value (see supplement).

**Figure 6:**
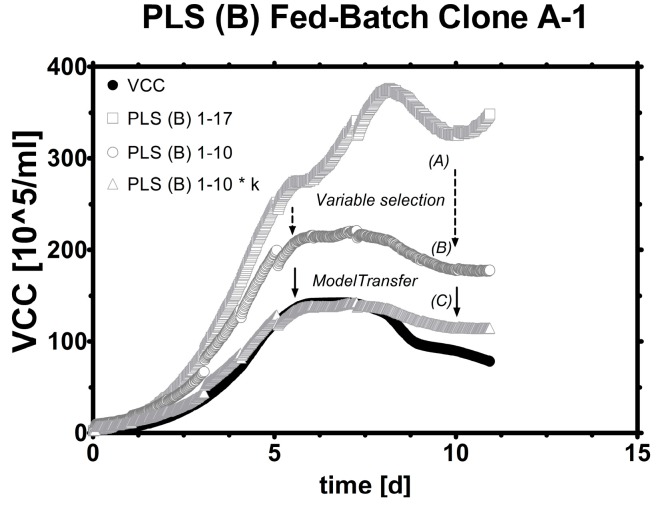
Transfer learning: (**A**) A PLS model (from Clone B) applied as it is for Clone A runs; (**B**) PLS (B) adapted by excluding the highest seven frequencies from the model; (**C**) PLS (B) modified with a linear factor. All runs were fed-batches with decreasing viability.

When this statistically linear factor was considered and applied by multiplication on the same dataset, R2 stayed the same, only the ratio of estimated *vs*. real VCC (the slope κ in Equation (4)) changed to one unit estimated VCC per one unit real VCC (Figure 6B,C). A PLS model constructed from a different cell line can be therefore readily re-used for any unknown cell line by knowledge of this simple linear and dimensionless factor termed Kappa (κ [-]), which is calculable online and offline due to its simple formula, where $\hat{y}$ is the estimated VCC, $\hat{x}$ the estimated VCC from a prior model, and d the intercept, all in (10^5^/mL).

(4) $$\hat{y}=\kappa \cdot \hat{x}+d$$

Because κ is calculable, it can be readily obtained autonomously already during the first bioreactor run of the new clone. This may be of importance for researchers or contract manufacturers with very tight timelines where no runs can be afforded for the establishments of “standard fed-batch profiles” and also where material is difficult to acquire or resources of any kind are a limiting factor. In our experience, the slope is available early in the process, when the culture is still in an exponential growth phase, but sometimes early process events such as pH regulation or aeration as well as drifts may lead to an inaccurate determination. Therefore, it is advised to start such an algorithm after a short lag time of a few hours (see supplement).

**Figure 7:**
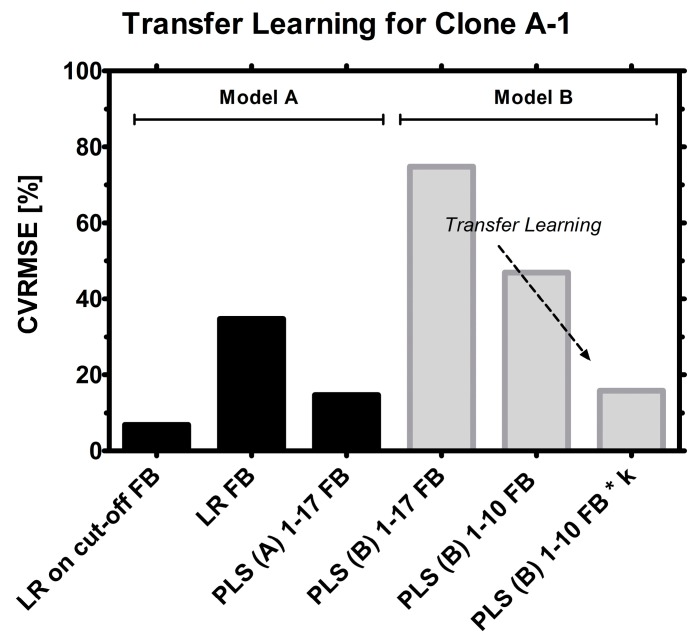
Model selection. From left to right: LR for highly viable fed-batch, LR for the whole FB, PLS regression for FB. Then, a Clone B model was employed to estimate Clone A: PLS model used as it is, PLS model with selected frequencies (1–10), PLS model with selected frequencies (1–10) and a linear factor (k).

This methodology has been conducted also vice versa by using a PLS model from the Clone A training data and applying the same calculation routine to Clone B fed-batches. The results are available and described later on in this paper as well as in the supplements. A step-by-step transfer of model B to model A runs was investigated under the previously defined model acceptance criteria. The transferred Clone B model performed well with roughly 20% CVRMSE (Figure 7) considering that a linear regression calibration of the same clone and model (A) was determined with roughly double CVRMSE (~40%).

If clone A was a new clone and used the first time in a bioreactor setting, naturally no runs would be available for constructing a PLS model for this cell line and standard linear regression would be the only available method for estimating VCC. PLS models from historical runs however may be useful for early biomass estimation where few or no data are available.

**Figure 8:**
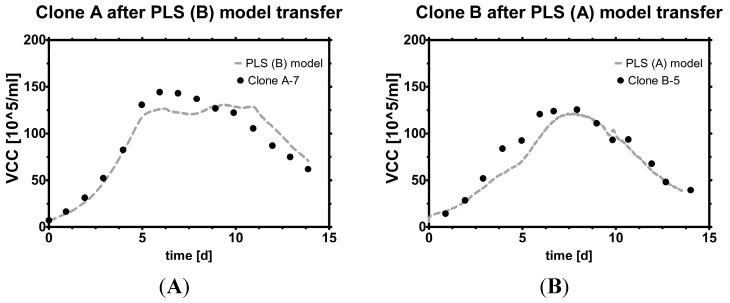
Model transfer. Estimation of VCC *versus* offline measurements with (**A**) PLS (B) model used on Clone A data; (**B**) PLS (A) model used on Clone B data. The death phase is captured sufficiently well with both models. The PLS (A) model seems to capture the decline phase slightly better in this particular run (see supplement for more runs), possibly because it was built with more data than the other model. Transferable, real-time resolved knowledge of VCC with this level of accuracy is believed to contribute significantly to the field of bioprocess monitoring and control.

Exemplarily, the power of the model transfer is shown on two examples: A PLS (B) model is transferred so that Clone A-7 data is estimated, and the same was done with a PLS (A) model for Clone B-5 data, the errors are shown later in Figure 8.

#### 3.4.3. Reasons for Transferability

The difference in the slope VCC *vs.* capacitance was observed by many research groups in the past [1,2,10,45]. The most common interpretations range from a change in size [5], a difference in internal or membrane conductivity [30] to the presence of mitochondria or other organelles [21]. Some researchers have observed that size alone cannot explain the difference in slope [8,12] of biological objects, or only after extensive offline de-correlation [35], which is not in the spirit of automatic process control. Therefore it is hypothesized that each clone exhibits a certain unique capacitance response to frequencies excitation in the right range.

### 3.5. Validation and Comparison with Literature

**Figure 9:**
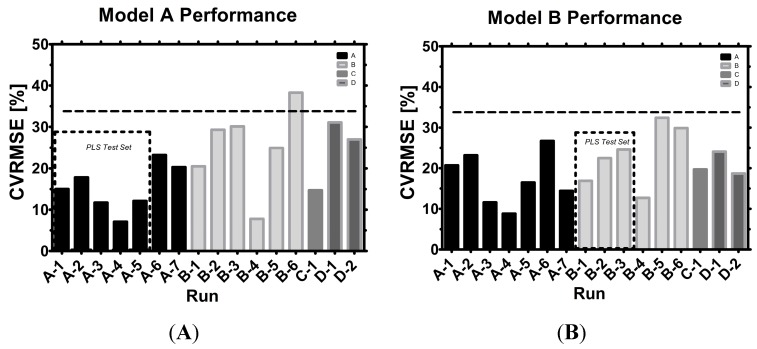
Model (**A**) *vs.* model (**B**) performance: The test sets for the PLS models are indicated, while all other runs served as model validation sets. A horizontal dashed line indicates 33% CVRMSE. Small differences in final CVRMSE calculation are possible when minute-by-minute records of capacitance spectra are aligned with the daily offline VCC measurements by various techniques [46,47,48].

Model A was validated with Clone B, C and D. Model B was validated with Clone A, C and D. For Clone A and B, PLS models could be constructed, while Clones C and D did not yield enough data. Model B contained only three runs from mixed locations and scales from Clone B while model A contained five runs from scale 1, indicated by a dotted black square. All remaining runs (with varying scales and clones) were used as validation datasets. A comparison of both models shows that almost all runs were below 33% CVRMSE, indicating a satisfying capability to predict VCC in a fed-batch with declining viability (Figure 9).

#### 3.5.1. Scale to Scale Transferability

Clone A was satisfyingly well predicted in either model with varying degrees of accuracy. In A7, a sudden burst of gas bubbles caused a slight drop in capacitances which was better predicted by model B, thus leading to a better final CVRMSE. Clone B runs were partially better predicted by model A (B4, B5), but one run (B6) also trespassed the acceptance criterion. However, in B6 the cells were treated with a detergent followed by a strong formation of cell debris which made it difficult to make use of frequency scanning in general. B5 experienced a strong morphological change in cell size, which caused the designated model to constantly overestimate larger cells. Clone C prediction was slightly improved when a model in the same scale was employed (model A). Model B, a mixed scale model, could be used to predict VCC sufficiently well. The difference in CVRMSE was rather small (5%) and the total CVRMSE did not exceed 20%, indicating sufficient accuracy in fed-batches. Clone D performed in general slightly better (5%–10% CVRMSE) with model B than model A. Both model predictions were sufficiently accurate for the previously defined acceptance criteria.

Conclusively, scale had a certain influence on model performance, but the influence was rather low in the given datasets of 16 runs.

#### 3.5.2. Clone to Clone Transferability

Four clones were analyzed via capacitance profiling. The emitted frequencies evoked slightly different amounts of capacitance, leading to a physiological or morphological link between the measurement and individual clone. By knowing the proportion of capacitance per clone, linear factors can be considered in the model generation. However, such proportions may not only depend on the clone itself. Morphological differences might be associated with differences in the physico-chemical environment of platform media and processes. Clone B was subject to very orthogonal process conditions leading to various physiological and morphological responses. The worst estimation of VCC thus happened in Clone B runs, where cell size increase correlated with overestimation of VCC (B5) and massive cell shrinkage correlated with underestimation (B6). Knowledge of the exact surface size of individual cells in real time together with a minimum of interfering particles might therefore lead to an improved model performance in any cell line.

In brief, either model predicted VCC well, no matter if the clone was A, B, C or D and it is possible that new CHO clones, and perhaps even other cell lines, follow this pattern.

#### 3.5.3. Internal Model Comparison

**Figure 10:**
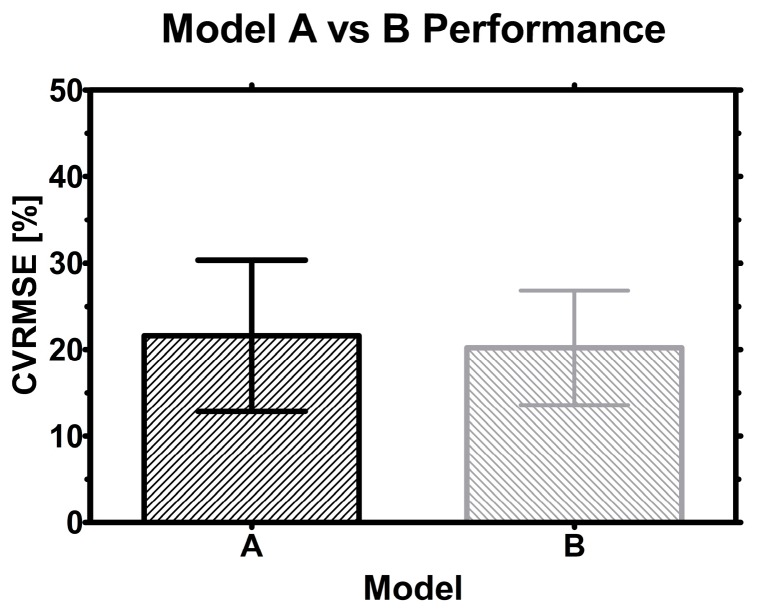
Model performance: Bars indicate the average CVRMSE found for model A and B, while error bars represent the standard deviation (SD) in the sample.

Average CVRMSE of both models were compared and plotted together with their corresponding standard deviation (σ). Because of the large variance (roughly 20% ± 9% σ for average) in both datasets no significant difference between the methods was found which emphasizes their interchangeable character and universal applicability (Figure 10). However, the hybrid scale model B had slightly less variance which might indicate slightly more consistency in VCC estimation.

The hybrid model might prove to be more robust with more runs, because the influence of scale may be further integrated in the statistical model. On the other hand, data from only one scale but orthogonal process conditions might be equally suited to estimate VCC in other scales or clones. Since statistical models rely on numbers, time and more data from either approach will tell which strategy works best in the end.

#### 3.5.4. External Model Comparison

A direct comparison between this contribution and the scientific work from other research groups is displayed below (Table 2). Due to the application of different performance criteria, post-processing steps, shorter observation windows (batch only) or other target variables than VCC (PCV) some data are not comparable. Although some authors provided the coefficient of determination (R^2^), caution is advised when interpreting it outside of the specified range (which is mostly the highly viable phase in cultures). Somehow, numeric CVRMSE values were hard to come by for mechanistic models in particular, which becomes apparent because all PLS and linear models had them included.

**Table 2:** Literature model performance.

Author	Year	CVRMSE	Comments	Ref
*Noll*	1998	n.a.	Linear model, R^2^ = 0.99, from a calibration curve with a serial dilution of a defined cell concentration	[8]
*Cannizzaro*	2003	9%–22% Batch phase, 24%–36% perfusion fed-batch	PLS model, 1 and 2 principal components, only one run available for validation (2 runs available), perfusion process with high viability	[12]
*Ansorge*	2007	n.a.	Linear model, 20% change in cell size corresponds to the third power (80% variance) in permittivity signal; R^2^ = 0.99 provided for batch phase	[10]
*Ansorge*	2010	n.a.	No numeric performance parameters from the Cole-Cole model available. Linear model parameters: R^2^ = 0.74–0.89, capacitance *vs.* packed cell volume (PCV), two different clones in a fed-batch, only samples with viability >70% taken into account	[1]
*Opel*	2010	7%–23% Mixed results, batch and fed-batch	PLS model, Result from cross validation with 5 principal components using 5 batches and 5 fed-batches as data source. Relative error is based on viable packed cell volume (vPCV)	[5]
*Heinrich*	2011	n.a.	No numeric performance parameters from the Cole-Cole model available. Linear model parameters: R^2^>0.98 for highly viable cells in perfusion	[45]
*Parta*	2013	5%–45% without smoothing, 9%–15% with smoothing, all fed-batch	Three principal components, using always 1 out of 6 fed-batches for validation, with and without Savitzky-Golay smoothing to compensate extreme outliers	[39]
*This contribution*	2014	7%–38% model A 9%–32% model B all fed-batch	Three principal components, one model (A or B) predicts VCC for 4 different clones and two different scales in a total of 16 orthogonal fed-batches	[-]

## 4. Conclusions and Outlook

This contribution proposes a roadmap for creating and validating a universal PLS model for biomass estimation in mammalian fed-batch cell cultivations with capacitance spectra. Transferable models were established and tested on a total of 16 fed-batches, comprising four clones and two different scales under highly orthogonal process conditions typical in process development.

The herewith presented novel approach is able to estimate viable cell concentration in real time completely without further off-line analytics after exactly one biomass measurement at inoculation for offset correction. Viable cell concentration from new clones, which were not tested in a bioreactor setting yet, can be estimated with these statistical models by calculation of a linear attenuation factor κ, which can be established while the process is still running. Because no performance criteria were defined in literature yet, a new acceptance criterion is presented, which is the CVRMSE of the monitored fed-batch run. CVRMSE itself does not require further pre- or post-treatment of the continuously recorded data, but an improvement in accuracy is expected when measured VCC is splined in-between measurements and compared to the continuously estimated model VCC [46].

A minimum of one dataset is required for creation and cross-validation of a PLS model, however, validation on the same test set tends to be too optimistic. Results from the model on new datasets are expected to improve when the PLS model is trained and validated with more than just one dataset, in this example at least five for model A and at least three for model B. A quickly growing database of fermentations with little process variation in production scale is expected to make one universal PLS model even more accurate and powerful, and will open up new avenues in bioprocess development.

## Supplementary Files

Supplementary File 1

## Abbreviations

*a*_1_ − *a*_17_: Capacitance 1–17 (pF/cm)
ANN: Artificial Neural Networks
B: Batch
FB: Fed-Batch
*c*_1_ − *c*_17_: Coefficient 1–17 [-]
CHO: Chinese Hamster Ovary
CVRMSE: Coefficient of Variation of RMSE (Root Mean Square Error)
*d*: Offset (cells/mL)
*fc*: Capacitance at the critical frequency (pF/cm)
FQ: Frequencies
κ: Linear factor [-]
LR: Linear Regression
mab: Monoclonal antibody
MC: Mean Centering
MLR: Multiple Linear Regression
*n*: Number of measurements
PC: Principal Component
PCR: Principal Component Regression
PLS: Partial Least Squares
PLS-R: Partial Least Squares Regression
R^2^: Regression Coefficient [-]
RMSE: Root Mean Square Error (cells/mL)
SD: Standardization
TCC: Total Cell Concentration (cells/mL)
VCC: Viable Cell Concentration (cells/mL)
$\hat{x}$: Estimated VCC from a prior model (cells/mL)
y: Measured VCC (cells/mL)
$\bar{y}$: Average VCC (cells/mL)
$\hat{y}$: Estimated VCC (cells/mL)

## References

[1] Ansorge S., Esteban G., Schmid G. (2010). On-line monitoring of responses to nutrient feed additions by multi-frequency permittivity measurements in fed-batch cultivations of CHO cells. Cytotechnology.

[2] Yardley J.E., Todd R., Nicholson D.J., Barrett J., Kell D.B., Davey C.L. (2000). Correction of the influence of baseline artefacts and electrode polarization on dielectric spectra. Bioelectrochemistry.

[3] Dabros M., Dennewald D., Currie D.J., Lee M.H., Todd R.W., Marison I.W., Stockar U. (2008). Cole-Cole, linear and multivariate modeling of capacitance data for on-line monitoring of biomass. Bioprocess Biosyst. Eng..

[4] Harris C.M., Todd R.W., Bungard S.J., Lovitt R.W., Morris J.G., Kell D.B. (1987). Dielectric permittivity of microbial suspensions at radio frequencies: A novel method for the real-time estimation of microbial biomass. Enzyme Microb. Technol..

[5] Opel C.F., Li J., Amanullah A. (2010). Quantitative modeling of viable cell density, cell size, intracellular conductivity, and membrane capacitance in batch and fed-batch CHO processes using dielectric spectroscopy. Biotechnol. Prog..

[6] Favre E., Voumard P., von Stockar U., Péringer P. (1993). A capacitance probe to characterize gas bubbles in stirred tank reactors. Chem. Eng. J..

[7] Yardley J.E., Kell D.B., Barrett J., Davey C.L. (2000). On-line, real-time measurements of cellular biomass using dielectric spectroscopy. Biotechnol. Genet. Eng. Rev..

[8] Noll T., Biselli M. (1998). Dielectric spectroscopy in the cultivation of suspended and immobilized hybridoma cells. J. Biotechnol..

[9] Zeiser A., Bédard C., Voyer R., Jardin B., Tom R., Kamen A.A. (1999). On-line monitoring of the progress of infection in .Sf-9 insect cell cultures using relative permittivity measurements. Biotechnol. Bioeng..

[10] Ansorge S., Esteban G., Schmid G. (2007). On-line monitoring of infected Sf-9 insect cell cultures by scanning permittivity measurements and comparison with off-line biovolume measurements. Cytotechnology.

[11] Ansorge S., Lanthier S., Transfiguracion J., Henry O., Kamen A. (2011). Monitoring lentiviral vector production kinetics using online permittivity measurements. Biochem. Eng. J..

[12] Cannizzaro C., Gügerli R., Marison I., von Stockar U. (2003). On-line biomass monitoring of CHO perfusion culture with scanning dielectric spectroscopy. Biotechnol. Bioeng..

[13] Niklas J., Heinzle E., Hu W.S., Zeng A.-P. (2012). Metabolic flux analysis in systems biology of mammalian cells. Genomics and Systems Biology of Mammalian Cell Culture.

[14] Chen Z., Chen Y., Chen J., Shen C. (1992). Effects of ammonium and lactate on hybridoma cell growth and metabolism. Chin. J. Biotechnol..

[15] Cruz H.J., Moreira J.L., Carrondo M.J. (1999). Metabolic shifts by nutrient manipulation in continuous cultures of BHK cells. Biotechnol. Bioeng..

[16] Templeton N., Dean J., Reddy P., Young J.D. (2013). Peak antibody production is associated with increased oxidative metabolism in an industrially relevant fed-batch CHO cell culture. Biotechnol. Bioeng..

[17] Zeng A.-P., Hu W.-S., Deckwer W.-D. (1998). Variation of stoichiometric ratios and their correlation for monitoring and control of animal cell cultures. Biotechnol. Prog..

[18] Ivorra A. (2002). Bioimpedance monitoring for physicians: An overview. Cent. Nac. Microelectròn. Biomed. Appl. Gr..

[19] Garthwaite P.H. (1994). An lnterpretation of partial least squares. J. Am. Stat. Assoc..

[20] Wise B.M. (2015). Properties of Partial Least Squares (PLS) Regression, and Differences between Algorithms.

[21] Lohninger H.H. (2000). Datalab 3.5, A Programme for Statistical Analysis. http://datalab.epina.at/.

[22] Rathore A.S., Mhatre R. (2011). Quality by Design for Biopharmaceuticals: Principles and Case Studies.

[23] Rosipal R., Krämer N., Saunders C., Grobelnik M., Gunn S., Shawe-Taylor J. (2006). Overview and recent advances in partial least squares. Subspace, Latent Structure and Feature Selection.

[24] Haenlein M., Kaplan A.M. (2004). A beginner’s guide to partial least squares analysis. Underst. Stat..

[25] Geladi P., Kowalski B.R. (1986). Partial least-squares regression: A tutorial. Anal. Chim. Acta.

[26] Tobias R.D. An introduction to partial least squares regression. Proceedings of the 20th Annual SAS Users Group International Conference.

[27] Mehmood T., Liland K.H., Snipen L., Sæbø S. (2012). A review of variable selection methods in Partial Least Squares Regression. Chemom. Intell. Lab. Syst..

[28] Pinto R.C.V., Marinho P.A.N., Oliveira A.B., Esteban G., Melo P.A., Medronho R.A., Castilho L.R., Noll T. (2010). Biomass monitoring and cho cell culture optimization using capacitance spectroscopy. Cells and Culture.

[29] Wong J. Implementation of Capacitance Probes for Continuous Viable Cell Density Measurements for 2K Manufacturing Fed-Batch Processes at Biogen Idec. http://www.infoscience.com/JPAC/ManScDB/JPACDBEntries/1394130144.pdf.

[30] Justice C., Brix A., Freimark D., Kraume M., Pfromm P., Eichenmueller B., Czermak P. (2011). Process control in cell culture technology using dielectric spectroscopy. Biotechnol. Adv..

[31] Beving H., Eriksson L.E., Davey C.L., Kell D.B. (1994). Dielectric properties of human blood and erythrocytes at radio frequencies (0.2–10 MHz); dependence on cell volume fraction and medium composition. Eur. Biophys. J..

[32] Gerckel I., Garcia A., Degouys V., Dubois D., Fabry L., Miller A.O.A. (1993). Dielectric spectroscopy of mammalian cells. Cytotechnology.

[33] Kell D.B., Woodward A.M., Davies E.A., Todd R.W., Evans M.F., Rowland J.J., Rzoska S.J., Zhelezny V.P. (2005). Nonlinear dielectric spectroscopy of biological systems: Principles ans applications. Nonlinear Dielectric Phenomena in Complex Liquids.

[34] Markx G.H., Kell D.B. (1995). Use of dielectric permittivity for the control of the biomass level during biotransformations of toxic substrates in continuous culture. Biotechnol. Prog..

[35] Ansorge S., Esteban G., Schmid G. (2010). Multifrequency permittivity measurements enable on-line monitoring of changes in intracellular conductivity due to nutrient limitations during batch cultivations of CHO cells. Biotechnol. Prog..

[36] David J., Nicholson D.B.K. (1996). Deconvolution of the dielectric spectra of microbial cell suspensions using multivariate calibration and artificial neural networks. Bioelectrochem. Bioenerg..

[37] Lohninger H. (1999). Teach/Me—Data Analysis.

[38] Beebe K.R., Pell R.J., Seasholtz M.B. (1998). Chemometrics: A Practical Guide.

[39] Párta L., Zalai D., Borbély S., Putics A. (2014). Application of dielectric spectroscopy for monitoring high cell density in monoclonal antibody producing CHO cell cultivations. Bioprocess Biosyst. Eng..

[40] El Wajgali A., Esteban G., Fournier F., Pinton H., Marc A. (2013). Impact of microcarrier coverage on using permittivity for on-line monitoring high adherent Vero cell densities in perfusion bioreactors. Biochem. Eng. J..

[41] Zeiser A., Elias C.B., Voyer R., Jardin B., Kamen A.A. (2000). On-line monitoring of physiological parameters of insect cell cultures during the growth and infection process. Biotechnol. Prog..

[42] Davey C.L., Markx G.H., Kell D.B. (1990). Substitution and spreadsheet methods for analysing dielectric spectra of biological systems. Eur. Biophys. J..

[43] Natschläger T., Zauner B. Fused Stage-Wise Lasso—A Waveband Selection Algorithm for Spectroscopy. http://www.scch.at/de/publikationen/publication_id/802.

[44] Torrey L., Shavlik J. Transfer Learning. ftp://ftp.cs.wisc.edu/machine-learning/shavlik-group/torrey.handbook09.pdf.

[45] Heinrich C., Beckmann T., Büntemeyer H., Noll T. (2011). Utilization of multifrequency permittivity measurements in addition to biomass monitoring. BMC Proc..

[46] MATLAB, Inc. Matlab 1-D Data Interpolation with Interp1. http://de.mathworks.com/help/matlab/ref/interp1.html.

[47] Motulsky H. (2004). Fitting Models to Biological Data Using Linear and Nonlinear Regression: A Practical Guide to Curve Fitting.

[48] Motulsky H. (2013). Intuitive Biostatistics: A Nonmathematical Guide to Statistical Thinking.

